# Supplementary material for: Patterns of Intron Gain and Loss in Fungi
Source: PLoS Biol. 2004 Nov 30;2(12):e422. doi: 10.1371/journal.pbio.0020422 (PMC532390; doi:10.1371/journal.pbio.0020422)
Supplement: Table S1 — Also available at http://genes.mit.edu/NielsenEtAl/. (4.3 MB ZIP). [file pbio.0020422.st001.zip › NielsenEtAl/html/1433.html]

AN6866.1.NCU07725.1.MG01710.1.FG09602.1


```
 CLUSTAL W (1.82) Multiple Sequence Alignments - Introns Inserted


Sequence 1: MG01710.1	294 aa
Sequence 2: FG09602.1	194 aa
Sequence 3: NCU07725.1	269 aa
Sequence 4: AN6866.1	267 aa
Alignment Length: 305 aa
Number Identitical Residues: 100 aa
Alignment Score (without introns) 5808


MG01710.1 	MKISSIVTLYMATVHALSNKLNRRSVTSMESLIDLSDANSALDLKQIRFQLI2R------
NCU07725.1	----------------------------MDAIIELSDVDKALDLSRIRYQLI2R------
FG09602.1 	----------------------------------MADAAHALDLARIRFQLM2LGTDSRS
AN6866.1  	----------------------------MDTAIDLSDASKALDLANIRFQLI2R------
          	                             .:  .::*.  **** .**:**:        

MG01710.1 	-LEDSITFHLIERVQFPLNK~NIYVSGAVKIPNSTLSFMDWCLWEQERVQSLIRRYESPD
NCU07725.1	-LEDTIIFHLIERVQFPLNK~NIYIPGAVPLPDTDLSLMDWYLWQQERLQSLMRRYESPD
FG09602.1 	RLEDTITFHLIERVQFALNS0TIYAPGAVELPESNLSFLDWYFREQEKLQSLIRRFESPD
AN6866.1  	-LEDTITFHLIERVQFPLNK~TIYIPGGVKIPNEQISLMDYLLRETERLQSRVRRYQSPD
          	 ***:* *********.**. .** .*.* :*:  :*::*: : : *::** :**::***

MG01710.1 	EYPFFPEALQSPILPSLNYPRILNPNDVNVNTKIKEFYTEHFLPAACPKPE-HGDRGAGQ
NCU07725.1	EYPFFPDAVQKPILESIDYPQILHPNNVNVNDQIKEFYTQKFLPSVCPDFG-REDRGVNK
FG09602.1 	EYPFFPDALQKPILKPLNYPRILYENDVNVNDKIKAFYTEKFLPAVCPDFG-REERGESQ
AN6866.1  	EYPFFPSALEKPILQPLDYPKILHDNDVNVNETIKTRYVQDILPAICPQFGGREDRGETQ
          	******.*::.*** .::**:**  *:****  **  *.:.:**: **.  .: :**  :

MG01710.1 	ENYGSSATADIACLQALSRRIHFGKFVAESKFRSEEKKYTEMIKAGDRQG~IGKAITNEA
NCU07725.1	ENYGSSATCDIACLQAISRRIHFGKFVAESKFQSETEKFTKYIQAGDREA~IGEAITNQA
FG09602.1 	ENYGSTATCDIACLQALSRRIHFGKFVAESKFRSEQEKYIRLIKAEDREG1S--------
AN6866.1  	ENYGSAATCDVSCLQALSRRIHFGKFVAESKFQKETEKFVALIKAGDRKE~IDEAITDAK
          	*****:**.*::****:***************:.* :*:   *:* **:   ..: :.  

MG01710.1 	VEKQVLDRLRLKVQTYGRDP-ITMSEHDIPSKINVEAVVSMYR~DYVIPLTKEVEVEYLM
NCU07725.1	VEKKVLERLKLKAETYGTDPSIGASEAESQRKINVEAVVAMYE~EFVIPLTKVVEVEYLM
FG09602.1 	-------------------------------------------~----------------
AN6866.1  	VEQKVLERLALKAKTYGTDP--GFPEQSGP-KIDVQAVQDMYK0EYVIPLTKVVEVEYLM
          	 ...  .    . .: . ..    .. .   . . .:       .   . :.  . .   

MG01710.1 	QRLQPAA--
NCU07725.1	QRLEPAPAQ
FG09602.1 	---------
AN6866.1  	QRLKGTQWE
          	.  . :  .
```
